# Subphrenic Abscess in Its Relations to Some Complications

**Published:** 1901-05

**Authors:** J. McF. Gaston

**Affiliations:** Atlanta, Ga.


### SUBPHRENIC ABSCESS IN ITS RELATIONS TO SOME COMPLICATIONS.*

BY J. McF. GASTON, JR., A.M., M.D.,
ATLANTA, GA.

Independent of the morbid changes in a known area, another syndrome of symptoms project themselves on the field, and we know we have a complication to deal with.

---

*Read before the Medical Association of Georgia, at Augusta, April, 1901.

In recent literature, appendicitis, Douglas's abscess and neurasthenia are associated with suppuration in the abdominal cavity. Pelvic complications are very difficult to differentiate from appendicitis, and at a recent meeting of the Southern Surgical and Gynecological Association, a large number of cases were reported. The discussion that resulted was especially interesting, in that the subject of appendicitis in the female was found to be a comparatively frequent complaint.

The relation between the occurrence of appendicitis and the presence of a neurasthenic tendency is also receiving attention.*

Subphrenic abscess is closely associated with empyema and pleurisy, and a close examination of the patient is never complete without palpation of the organs of the abdominal cavity, as well as auscultation and percussion of the chest.

The perception of fluctuation is a most important aid in diagnosis. In hepatic abscess a great point is gained when a peculiar wave-like motion can be detected at the point of the finger held between the eighth and ninth ribs, and a tap or two is given with the fingers of the other hand at the umbilicus.

J. Marion Sims has called attention to this, and to the peculiar melancholia associated with incipient disease of the liver resulting in abscess, in remarks made by him before the Medical Society of Virginia, October 25, 1879.

The diagnostic points depend, in a great measure, upon a very careful observation of each symptom as it arises, and a coordination of the symptoms pointing to a hepatic abscess. All of these troubles are not so difficult to discover alone, but when combined may be a source of considerable perplexity to the surgeon.

Two cases of some unusual complexity have been operated upon by me. A short abstract of one will be given, as it has been published in the *New York Medical Record*, under the title of

"SUBPHRENIC ABSCESS AS A COMPLICATION OF APPENDICITIS."

On June 3, 1900, I was asked by Dr. J. W. Carmichael to see W. E. F., aged 32, and examine him with reference to pain in region of appendix. Dr. W. Monroe Smith had seen him when he

* Page 256, *Philadelphia Medical Journal.*

was first brought home, and administered a hypodermic of morphia gr. ¼, with atropine gr. 1-150. At 7 A.M. his temperature was 100.2° F., respirations 26, pulse 100. His general appearance gave evidence of a strong, robust man, prostrated by some serious complaint. A prescription of ungt. belladonnæ oz. 1; pulv. camphoræ—Sig. Apply to abdomen, and cover with oiled silk—was given. He had already received a large dose of calomel, which was now followed by a tablespoonful of Epsom salts. His condition betokened a fulminating appendicitis, and he was accordingly watched carefully, his temperature, pulse, and respiration noted every half-hour. All preparations for an operation were made. He received morphia sulphate gr. ¼, with atropia sulphate gr. 1-150, hypodermically. His pulse was accelerated, and pain became intense. Two tablespoonfuls of castor oil, with spirits of turpentine one teaspoonful, were administered without result. The operation was performed in the afternoon, with the assistance of Drs. Smith, Carmichael and Gaston, Sr. A. C. E. mixture was administered. The parts were shaved, cleansed with soap and water, solution of carbolic acid and ether. An incision was made one and a half inches long over the tumor in the right iliac region, near the border of the rectus muscle. It was enlarged later to nearly three inches. The fibers of the transversalis and rectus muscles were separated by the fingers, and in the aponeurosis an aperture was made for a directory to guide the scissors in cutting. The peritonum was opened in same way. A small amount of dark fluid was found. A large piece of omentum was ligated with cat-gut and removed. The appendix was tied with silk, and cut with scissors. A portion of tissue near the appendix was excised, the stump treated with carbolic acid, and replaced. The appendix was gangrenous, and contained one enterolith. The peritoneum was wiped with peroxide of hydrogen, irrigated with normal salt solution and hot water. The wound was then closed except at lower angle, to allow for gauze drainage. Patient was placed in bed, and received a hypodermic of strychnia nitrate gr. 1-30. At 7:30 P.M. temperature 101, pulse 95, respirations 21. The ointment was continued. No morphine or water was allowed. Another hypodermic of strychnia nitrate gr. 1-30 was given at 11:30 P.M., when his temperature was

99.8, pulse 105. A quantity of bile was vomited, the respirations were hurried, and at 10 P.M. his pulse was 95, and temperature 100.2. He received every hour a tablespoonful of the following: Lime water, 3 oz., peppermint water, 1 oz., camphor water, 2 oz. Diffuse peritonitis seems to have existed. The tongue was red and raw, while the respirations were irregular, accelerated and spasmodic. The pulse was the chief danger-signal. Frequent dressings were used, and free drainage secured. Occasional small doses of calomel were given with resulting free discharges and relief. Sterilized gauze was used as a drain from the stump. Dioxide of hydrogen was continued for four weeks. Sublimate gauze was substituted for sterile gauze on the third day. Dressings were made twice daily.

Iodoform gauze was packed into the wound on the tenth day. Upon remembering the fluctuation in the sub-diaphragmatic region it was thought necessary to reopen the wound and drain. A complication of subphrenic abscess was evident. The pyogenic membrane and pus finally escaped at a dressing. Milk was allowed as nourishment. Chloretone internally and locally for pain. Tonics, like elixir of iron, quinine and strychnia, tincture of cinchona comp. and strychnia nitrate tablets were given. Enema of magnesia sulphate to obtain movement of bowels.

A large colon tube was used very satisfactorily.

The above complication in appendicitis is rare. Baldwin's cases make the forty-fourth and forty-fifth. Many cases were not diagnosticated until after death. Pathological examination shows an intra- and extraperitoneal variety.

In Baldwin's cases the first operation was performed on June 6, 1899, and not until July 20, 1899, did the abscess appear. After incision immediately below the twelfth rib an enormous amount of pus from a large cavity with ragged edges escaped. The cavity extended up toward the diaphragm above the liver, and closed in six weeks.

The second case was operated upon on July 28, 1899, and again on August 28, 1899, for abscess. The cavity was about the size of an orange, somewhat ragged interior, which was washed out with a counter opening for drainage. It was packed with gauze, and closed in two weeks.

In my case the symptoms were, evidently from the start, due to abscess because of fluctuation and rapid breathing. A channel, so to speak, was opened by the cutting away of the adherent omentum during operation for appendicitis. The discharge came from direction of the diaphragm, as evidenced by an increase when patient coughed, or on deep inspiration. The outcome was peculiarly fortunate, not requiring another operation. As a probable cause of this abscess, the patient gives a history of injury of this region some months before.

The other case was one of hepatic abscess, which was aspirated, and seemed to be relieved temporarily when symptoms of empyema seemed to appear, and death followed. These were men.

Another still was in the person of a lady, and has not been reported. Drs. J. C. Olmsted and J. McFadden Gaston treated this case. There was an abscess in Douglas's cul-de-sac, complicated with abdominal organs. Free irrigation and antiseptic treatment resulted in a cure in this lady.

But before the abscess ruptured grave doubt existed as to its origin, whether in the appendix, or whether the pus came from the tube.

In a patient which I attended some years ago, a similar abscess was discharged through the uterus after every symptom of gall-bladder obstruction with jaundice and finally typhoid fever. She recovered entirely, and afterward bore a healthy child, and never had a recurrence.

It is well to consider the presence of retroperitoneal abscesses, when an incision in the lumbar region has been suggested, as in these cases the dangers arising from peritonitis, and a migratory infection, are less than when the peritoneal cavity is invaded. It is evident that in the great diversity of conditions we should consider carefully, and, I might say, prayerfully, each individual case.

At times simply a tuberculous deposit is found on the appendix, and then we have very little more to do than to excise this diseased portion of the appendix. But in the great majority of cases an enterolith is encountered, and there is a process of gangrene which begins in the mucous membrane of the appendix and spreads to the confines of the mesentery, and ends in perforation of the muscular and serous coats of the appendix.

As appendicitis is a disease associated with so many complications, is it not wise to investigate thoroughly the nervous system, the lymphatic system, and the digestive and alimentary canal, before determining the means of relief to be adopted?

All pains in the region of the appendix are not due to appendicitis, and it is equally true that we may have a long-continued abscess of the liver without any apparent enlargement of the liver or pain.

In the paper of Dr. J. Marion Sims, which has been already mentioned, he details a case in which the eminent neurologist, Dr. William A. Hammond, had formed a diagnosis of abscess of the liver from the symptoms of hyperemia of the brain, and had afterwards made a physical examination which was not entirely confirmatory, and which sufficed to cause an exploratory aspiration, with the evacuation of a quantity of pus (fifteen and a half ounces). The general health and mental condition of the patient at once improved, and he remained well after this, suffering only from previous paralysis.

"Dr. Hammond aspirated the liver twenty-six times in the last two years," said Dr. Sims in 1879. One of his patients was Dr. E. S. Gaillard, and nearly all the cases were in men who had undergone great mental exertion. In fifteen cases he evacuated abscesses and effected cures. In eleven cases the operation was unsuccessful, but attended with no ill effects whatever. Dr. Hammond was in the habit of passing the needle through the intercostal space between the eighth and ninth ribs, at a point about an inch in advance of a line drawn from the axilla to the pelvis. Drs. Emory Lanphear,* W. E. B. Davis,† Joseph Price,‡ W. Gill Wylie, and many others, as early as 1890, endorsed Tait's ideas of operative treatment for peritonitis, having first used saline cathartics in great quantities. In suppurative peritonitis particularly is an operation indicated with the evacuation of pus, wherever located.

* Kansas City *Medical Index*, July, 1890.
† Read before Medical Association of the State of Alabama, April 13, 1890.
‡ *Medical News*, August 9, 1890.

The earlier the operation can be undertaken in all such cases the better are the chances of recovery. I wish to give some of my own personal observations in this couple of rare cases.

One symptom that may prove of distinctive importance bearing upon a diagnosis between hepatic abscess and subphrenic abscess, is that the diaphragm may be pushed upward in hepatic abscess, while in subphrenic abscess proper we may have the stomach pushed down, and the intestines pressed forward. The most common and striking accompaniment of suppuration in any part of the body is variable temperature and rigors.

In one case reported by Lees and Pepper, the temperature had reached 106°; the symptoms pointed to an abscess near the stomach, but upon incision as in gastrostomy no pus was found, but the stomach had a lymph matting its walls to the intestines.

It was by aspiration in the seventh interspace, on the left side, that pus was discovered, and this was found to come from below the diaphragm which had been penetrated by the needle.

In hepatic abscess we usually find hurried and abdominal respiration; while in subphrenic abscess there is slower respiration, but it is mostly confined to the thorax.

The pulse rate, or rather heart-beat, per minute, is slower than normal in hepatic abscess, owing to great pressure upward upon the heart, and to the left upon the abdominal aorta and the iliac vessels.

In hepatic abscess we may notice a displacement of the right lobe of the lungs, but in subphrenic abscess, more particularly, the stomach, spleen and kidneys. We may have no means of differentiating these two affections, as to their side, as we have in appendicitis. But right hepatic abscess is far more commonly met with than abscess of the left lobe of the liver.

Empyema is usually unilateral, but we may have double empyema. At the same time in empyema we will usually find, when it occurs on either side, a destruction of the vocal fremitus on the side affected; and displacement of the heart when empyema is on the left side. The complication of the two conditions must also be looked into, as pus may have burrowed through the diaphragm.

It is an important diagnostic device in empyema to observe the influence of gravity upon the collection of fluid in the pleural cavity. In the event the pus gravitates to the left side when the patient is put on that side, and no dullness exists on the right side, the strong presumption would be in favor of a unilateral empyema of the left pleural cavity.

When we see no change wrought by lateral position, or by placing the patient in an upright posture, then we suppose there may be a confusion arising from two sources, viz.: (1) A tense and unyielding pyogenic membrane with adhesions of the pleura; and, again, (2) a consolidation of lung.

Subphrenic abscess, we are told by Curtis, has been traced to every organ of the abdomen. Curtis * gives a slightly different method of differentiating affections of the liver, lungs and pleura from subphrenic abscess. He advises that in percussion and auscultation the examining physician note whether there is a concavity upwards or downwards in the area of dullness, and naturally suggests that the diaphragm will be domelike in subphrenic abscess from pressure upward, and a reverse condition or shape, by pressure downwards in empyema.

*Treatment.*—Subphrenic abscess should be treated by early incision and drainage. Aspiration in this locality is not a safe procedure on account of the dangers of perforating the hollow viscera. The organ that gave rise to the abscess may be examined, and if any ulcer has perforated the stomach or abdomen, the opening should be closed by suture.

Advantage of free drainage after operation is greater from the elevation of the head and shoulders and hips. This process is now very highly endorsed by Fowler, in his treatise on "Appendicitis" in his revised edition.

Normal salt solution and peroxide of hydrogen are useful agents in irrigation of the abscess cavity, both during and after the operation for evacuation of its contents.

Captain W. C. C., aged fifty-six, came from Montgomery, Ala., during a bad epidemic of yellow fever.

Abscess of the liver was suspected by his physicians, Drs. Wil-

* Twentieth Century Practice of Medicine, Vol. VIII, pp. 477-8, 9 and 480.

kinson and J. B. Gaston. In the effort to refugee from yellow fever he came to Atlanta, and a letter from Dr. J. B. Gaston showed that it had been their intention to aspirate. He was put under the care of my father, Dr. J. McFadden Gaston, and myself, October 21, 1897.

Upon examination the liver was found very much enlarged, and a diagnosis of hepatitis was formed. With the strong hope of relieving his immediate symptoms we put him on a prescription of

Massæ Hydrargyri....................................grs. 20.
Podophyllin.........................................grs. 2.
Pulv. Rhei..........................................grs. 30.
M. Divide into capsules No. 10.
Sig.: Take one every other night.

Another prescription, for external use, was given at the same time:

℞ Ung. Hydrargyri,

Ung. Belladonnæ,

Ung. Iodidi,

Ung. Camphoræ, } aa.................................ʒ 2.

M. Sig.: Rub well under margin of ribs. Cover with oiled silk.

On October 23 he was quite restless, and was given a combination of bromides of sodium, ammonium, potassium, lithium and calcium in teaspoonful doses in water.

Next day his pulse was 48 and very weak. Chest measured 16½ inches on the right side, in the semi-circumference from the ensiform cartilage. Temperature was 100.4°. The left side measured 17 inches, as described for the other side, a total circumference of 33½ inches, and three inches lower 15 on right and 18 on left; total 33.

It will be observed that the bulging occurred below the ensiform cartilage and very similar to a case of subphrenic abscess. (Stockton.)

On October 26 the temperature was 100.2 at noon; and at 5 P.M. 100.6°; pulse 72. Measurement of left side as before, 17¾ inches; right side 17. Three inches below measured 18 and 17½ respectively. A greater distention on the left side was somewhat unexpected, but might be accounted for by pressure of organs to that side by the pus situated on the right side.

On October 28 the following alterative was prescribed:

℞ Syr. Trifolium Co. (Parke, Davis & Co.)................℥ 3½.

Liquor Arsenii et Hydrargyri Iodi., F. (Donovan's So.) ʒ 4.

M. Sig.: Take a desertspoonful three times a day.

He was also directed to be nourished with Horlick's Malted Milk. As an additional hepatic derangement continued, and profuse sweating occurred at night on November 5, the use of the following was indicated:

℞ Acidi Nitro Hydrochlorici..............................ʒ 2.

Aquæ distillatæ, q. s. F.......................................ʒ 2.

Sig.: Take a teaspoonful in water every six hours.

On November 13 the following combination was given for aiding expectoration, and as a general stimulant to the respiratory organs:—

℞ Ammonii Carbonatis.................................. ʒ 1
Olei Terebinthinæ......................................fl ʒ 1
Syrupi Tolutani ...........................................fl ℥ 1
Aquæ Camphoræ..........................................fl ℥ 2
Mucilag Acacie, q. s.....................................fl ℥ 6
M. Sig Take a tablespoonful every two hours.

On November 20 his temperature was 100°, pulse 90. Breathing clearer on both sides.

A.M., temperature 99°, pulse 120, respiration 36. P.M., temperature 99°, pulse 110, respiration 32. The right side was somewhat dull. Flatness was noted on percussion of the left side.

On this day a prescription, to combat the symptoms of pain and difficult breathing, was given as follows:

℞ Hydrarg. Chloridi Mitis............................ grs. 1
Pulv. Ipecac et Opii...................................... grs. 30
Quiniæ Sulphatis........................................ grs. 30
Pulv. Camphoræ.......................................... grs. 1
M. Divide into capsules No. 12.
Sig.: Take one every night and morning.

After these were given his condition seemed better, with a normal temperature; especially was the left lung clearer of mucus. No liver dullness below ribs. Puncture of aspirator under ribs had healed, and at 5:15 P.M. temperature 99.6, respiration 32, pulse 96.

November 22, 5 P.M., temperature 98.7, pulse 90, respiration 32.

November 22, 12:30 P.M., temperature 98.4, pulse 100, respiration 34.

When asleep his respiration dropped to 19. He sweated a good deal. At one time his respiration was 18 while asleep. The tenderness over the median line is considerable.

Percussion reveals dullness over the median line. No pain or suffocation was noted in the region of the lungs.

November 23.—His weak condition necessitated an effort to build up his system, and he was put on Anæmiol in teaspoonful doses with hot water. November 23, 5:30 P.M., temperature 98, pulse 90, respiration 36.

At 10 P.M. his temperature was 98, pulse 80, and respiration 40.

On November 24, at 1 P.M., his pulse was 60, and weaker, his temperature 97°, while the respiration was not taken. The temperature, later in the afternoon, was 96.8, pulse 80, and respiration 40.

November 25, 2:30 A.M.—Being called out at this hour of the night his temperature was not taken, but his pulse was 100 and feeble, respiration 48. A teaspoonful of Anæmiol was administered; also a hypodermic injection of 1-25 grain strychnia nitrate with 1-25 grain of morphine sulphate. Before leaving his respiration had been reduced to 20 per minute. He died at 6:30 A.M.; his respiration had again reached 48; while temperature was 95, when seen shortly before the end. His condition was very much improved by the relief of pressure afforded by an operation performed two weeks before his death.

Aspiration was first performed in the region of the liver below the ribs by my father, Dr. J. McFadden Gaston. The aspirating needle was clogged with blood, and no distinct evidence of whether the needle touched the focus of pus was obtained. He was put under the anesthetic for the second time during the hour, and I inserted the needle into the interspace between the eighth and ninth ribs when a quantity of pus was drawn off—estimated at a pint and a half.

The pus was examined microscopically by Dr. F. S. Bourns, who was then pathologist of the Southern Medical College. The evidence of liver cells was sufficient, and various streptococci were found in the pus.

Subphrenic or sub-diaphragmatic abscess is, as the name implies, an abscess forming below the diaphragm. It is not the same as hepatic abscess, originating in the liver, or of suppurative peritonitis, arising from the serous membranes of the peritoneum. At the same time any of these conditions may result in a subphrenic abscess, or at least be complicated with one. There may also be the collections of pus above the diaphragm that complicate a case of subphrenic abscess. It is my object to investigate the subject of subphrenic abscess and these complications.

John J. Wishard,* of Indianapolis, Ind., has treated of the subject of hepatic abscess, and defined it as hepatitis, terminating in suppuration, due, probably in all cases, to microbic invasion, and in most cases to infection by way of the portal vein. Dr. T. H. Manley in a note upon the same subject says: "Dr. Wishard submits a valuable brochure on above topic, though there is one phase of the subject we would have been pleased to have seen further considered; this bears on the symptomatology, especially in differentiating between interstitial, true abscess of the liver, and subphrenic; and those of pleurisy and hepatic suppuration. Attention is called to this aspect of the case, here, for the reason that the writer, during the past year, saw three cases of hepatic suppuration, in all of which the primary symptoms pointed to pleuritic trouble. In one, diagnosis was only made on autopsy; in another the case was turned over to the surgeon for an external under operation for empyema; in the third the onset, and all the primary symptoms were those of pleural invasion. M. Dieulafoy, not long since, in the *Gazette Medicale*, considers at length the manner of pyogenic invasions here, and notes that it most commonly proceeds from a diseased appendix, through the lymphatic or portal vein, or direct infection up through the ducts from the duodenum."

Pierre Martin, of France, has written a work which is reviewed in the *Journal de Medicine et Chirurgie Prat.*, September 10, 1892. It is entitled "Subphrenic Pyothorax." He says: "It can exist as a purulent encysted collection in the superior part of the ab-

* *Medical Times and Register*, page 76, March, 1901.

dominal cavity, situated immediately below the diaphragm. In certain cases these collections contain gas, which may give a series of symptoms recalling pneumothorax. In the majority of cases, more than one-third of the cases known, this pyothorax was met with in patients with ulcers of the stomach or duodenum.

"It is to be noticed that duodenal ulcer and ulcer in the vicinity of the pylorus give rise to these purulent collections situated under the right half of the diaphragm. Ulcer of the stomach will cause left subphrenic abscess. The other causes most frequently observed are typhlitis, hydatid cysts, perimetritis, etc.

"But it is not necessary in case of gastric or intestinal lesion that there be perforation to produce the abscess; the neighborhood of the lesion is sufficient.

"The symptomatology of this lesion is often obscure. There are three periods which may be distinguished in the development of subphrenic pyothorax: the prodromic period, the period of invasion, and the period of stasis or thoracic period. In the first period the symptoms are those of the affection which may give rise to the subphrenic abscess. Vomiting and nausea after meals, frequent hematemesis, pains in the epigastric or hypochondriac regions.

"There is nothing in this period which would point to a dullness immediately under the diaphragm, unless there are some febrile symptoms that may be overlooked.

"In the second period the onset is sudden, although preceded by symptoms of peritonitis, due to perforation, or a purulent discharge by the intestine.

"Pain is the first sign to manifest subphrenic pyothorax; atrocious pain extends over the whole abdomen, without enabling the patient to tell the exact point of departure. It persists for some time, radiating to all parts; at the same time the respiration is not really interfered with, which is an important symptom for the diagnosis with pleurisy."

The further description given by Martin embraces the third period, with deformity of abdomen, with tympanites, tension and tenderness. The respiration is of the superior costal type.

A spontaneous opening through the diaphragm, pleura, or bronchial tubes is a termination attended with relief in some cases. Perforation of intestines may occur.

In regard to empyema following subphrenic abscess, there is a very interesting case mentioned in the *International Medical Magazine* for May, 1892. Charles G. Stockton conducted the medical part of the treatment and Dr. Roswell Park the surgical measures.

The subphrenic abscess appeared first, was opened and drained. Later an encysted empyema was discovered and aspirated. Nearly a pint of pus was evacuated. The resection of a section of one and a half inches in length of the eighth rib was performed. Three pints of pus were found at the time of the operation.

At one time his pulse was 106, respiration about 36 and temperature 102°. He recovered completely.

Levison, of Copenhagen, has reported a case of subphrenic abscess in which a puncture of the eighth costal interspace reached no pus, as the diaphragm had been pushed up, but a puncture in the seventh emptied gas and pus. There was an abscess cavity below the diaphragm, with no communication with the pleural cavity. This man's temperature reached 40° C (104° F), while his pulse beat was 150, and respirations were 36 per minute. In the year 1874 this case occurred and was published in 1876, even antedating Leyden's cases published in 1877. The slow course of a disease of this kind is evidenced by the date of onset being referred back to January, 1874, and ending in death September 22.

### A CASE OF COMPLETE PLACENTA PREVIA.

Frederick Coggeshall (*Boston Medical and Surgical Journal*, February 14, 1901) reports a case in which the patient was apparently *in extremis*. He at once started the subcutaneous injection of decinormal salt solution under both breasts and underneath the loose skin of the back, at the same time, by the means of a three-way tube. The foot of the bed was raised, and a grain 1-10 strychnine nitrate was given subcutaneously. Version and delivery were rapidly accomplished. The patient had stopped breathing, and artificial respiration, including the Laborde method, was applied. The uterus received a hot douche of 1:1000 bichloride solution as soon as it was empty, and was packed with gauze on which had been emptied a two-ounce bottle of liquor ferri chloridi. It contracted at once, and the hemorrhage was all over in about two minutes. The mother slowly recovered.—*Obstetrics.*

